# From population connectivity to the art of striping Russian dolls: the lessons from *Pocillopora* corals

**DOI:** 10.1002/ece3.3747

**Published:** 2017-12-27

**Authors:** Pauline Gélin, Cécile Fauvelot, Lionel Bigot, Joseph Baly, Hélène Magalon

**Affiliations:** ^1^ UMR ENTROPIE (Université de La Réunion, IRD, CNRS) Université de La Réunion St Denis La Réunion; ^2^ Laboratoire d'excellence‐CORAIL Perpignan France; ^3^ UMR ENTROPIE (IRD, Université de La Réunion, CNRS) Centre IRD de Nouméa Noumea New Caledonia; ^4^ Present address: Université Côte d'Azur CNRS Nice France

**Keywords:** Bayesian assignments, cluster, DAPC, microsatellites, *Pocillopora*, scleractinian

## Abstract

Here, we examined the genetic variability in the coral genus *Pocillopora*, in particular within the Primary Species Hypothesis PSH09, identified by Gélin, Postaire, Fauvelot and Magalon (2017) using species delimitation methods [also named *Pocillopora eydouxi/meandrina* complex *sensu*, Schmidt‐Roach, Miller, Lundgren, & Andreakis (2014)] and which was found to split into three secondary species hypotheses (SSH09a, SSH09b, and SSH09c) according to assignment tests using multi‐locus genotypes (13 microsatellites). From a large sampling (2,507 colonies) achieved in three marine provinces [Western Indian Ocean (WIO), Tropical Southwestern Pacific (TSP), and Southeast Polynesia (SEP)], genetic structuring analysis conducted with two clustering analyses (structure and DAPC) using 13 microsatellites revealed that SSH09a was restricted to the WIO while SSH09b and SSH09c were almost exclusively in the TSP and SEP. More surprisingly, each SSH split into two to three genetically differentiated clusters, found in sympatry at the reef scale, leading to a pattern of nested hierarchical levels (PSH > SSH > cluster), each level hiding highly differentiated genetic groups. Thus, rather than structured populations within a single species, these three SSHs, and even the eight clusters, likely represent distinct genetic lineages engaged in a speciation process or real species. The issue is now to understand which hierarchical level (SSH, cluster, or even below) corresponds to the species one. Several hypotheses are discussed on the processes leading to this pattern of mixed clusters in sympatry, evoking formation of reproductive barriers, either by allopatric speciation or habitat selection.

## 1 INTRODUCTION

Studying population genetic connectivity is first a matter of knowing what we work on, that is, accurately delimiting the evolutionary units. Indeed, the populations among which we want to assess the exchanges of alleles must be composed of individuals that belong to a unique and same species, in order to estimate genetic distances among comparable entities. That being said, it seems trivial, but the increasing discovery of highly divergent populations or divergent clusters among populations reveals the presence of possible cryptic species, somehow by serendipity, that the sole use of traditional taxonomic characters may have not highlighted. So population genetics data collected for estimating connectivity, and more broadly phylogeographic analyses or barcoding, may in turn be used to refine taxonomic knowledge at the species rank, making them hardly ineluctable in an approach of integrative taxonomy. As an illustration, trying to understand the biogeographic shift in the kelp *Lessonia nigrescens*, Tellier, Meynard, Correa, Faugeron, and Valero (2009) identified two cryptic species using a combination of four genes among 1,000 individuals covering more than 2,500 km of coastline. The two divergent genetic lineages show a parapatric latitudinal distribution: one extends from southern Peru (17°S) to central Chile (30°S), and the other from central Chile (29°S) to Chiloe Island (42°S), both lineages spatially overlapping in a narrow area (29–30°S) in discrete patches where individuals belong to either the northern or southern species. Likewise, studying the ecological interactions between a coral host and its crustacean exosymbionts, Rouzé et al. (2017) used barcoding methods to identify the exosymbionts and found two cryptic species in the shrimp *Alpheus lottini*, revealing the key role of cryptic diversity in structuring communities of mutualists and the importance of taking into account this diversity in ecological studies to better perceive the complexity of ecological processes. Similarly, Souter, Henriksson, Olsson, and Grahn (2009) studied the connectivity pattern in the coral *Pocillopora damicornis* in East Africa and, after identifying two cryptic lineages using mitochondrial markers, chose to analyze them separately. Also, exploring the species diversity of the hydrozoans from the Aglaopheniidae family, Postaire, Magalon, Bourmaud, and Bruggemann (2016) revealed extensive lineage diversity and cryptic species in two common species, *Lytocarpia brevirostris* and *Macrorhynchia phoenicea*. Then, studying the connectivity of one of the cryptic species within the *L. brevirostris* complex using microsatellites, Postaire, Gélin, Bruggemann, and Magalon (2017) found a high genetic differentiation among populations, each island housing an independent evolutionary lineage, probably representing different species. *In fine*, two populations that have diverged enough can be considered as distinct units (e.g., species) on which, several studies, for example, genetic structuring and connectivity, environmental responses in face of perturbation, and conservation plan, will be set up. Nevertheless, the speciation process is slow and gradual. Thus, it is sometimes tricky to put a frontier between different units, notably when the speciation process is not achieved (De Queiroz, 2007), that is, in the gray zone starting from one species and ending to two new ones.

In marine systems, a large number of studies have shown that species with no or low dispersal (often linked with larval phase) tend to present significant genetic structure over small spatial scales while high dispersal abilities are not correlated with population subdivisions (Kelly & Palumbi, 2010). In general, high dispersal species present large population sizes, huge ranges, and rapid gene flow: characteristics that should slow species formation, confirmed by fossil data (Jablonski, 1986). Nevertheless, high dispersal potential does not always lead to high gene flow because of selection (Hilbish & Koehn, 1985), local genetic drift (Reeb & Avise, 1990), and complex homing behavior (Baker et al., 1990). Indeed, habitat preferences could lead to segregate individuals and promote divergence till sympatric speciation even with remaining gene flow (see for reviews, Pinho & Hey, 2010 or Bowen, Rocha, Toonen, & Karl, 2013). Among marine organisms, scleractinians represent a good example of species with potentially high dispersal [e.g., larval lifetime of 30 days for *Heliopora coerulea* (Harii, Kayanne, Takigawa, Hayashibara, & Yamamoto, 2002), 100 days in *Pocillopora damicornis* (Richmond, 1987), and >200 days in some species from the genera *Acropora*,* Favia*,* Goniastrea*,* Monstastrea* (Graham, Baird, & Connolly, 2008)], but their dispersal and settlement are constrained by several biotic and abiotic factors (hydrodynamics, light, temperature, gravity, surface texture, or presence of conspecifics; Rodriguez, Ojeda, & Inestrosa, 1993).

Like almost all the morphospecies from the genus *Pocillopora, P. eydouxi* has been described widely distributed in the Pacific Ocean and Indian Ocean, and the Red Sea, but absent from the Atlantic Ocean. Some recent studies have revisited *Pocillopora* taxonomy in light of molecular data. Using species delimitation methods based on mitochondrial markers, Gélin, Postaire, Fauvelot and Magalon (2017) found that *P. eydouxi* forms a primary species hypothesis [PSH, *sensu* Pante et al. (2015)], named PSH09 therein [see Gélin, Postaire, et al. (2017) for the name correspondence with the other studies]. This PSH corresponded to the complex *P. eydouxi/meandrina* ( *sensu* Schmidt‐Roach, Miller, Lundgren, & Andreakis, 2014) and will be further named *P. eydouxi* or PSH09 to make the reading easier. Then, performing assignment tests on multilocus genotypes (257 colonies and 13 microsatellites), they further revealed the occurrence of three secondary species hypotheses [SSH, *sensu* Pante et al. (2015)]: SSH09a is restricted to the Western Indian Ocean, and SSH09b and SSH09c are found in sympatry but restricted to the Pacific Ocean. Generally, colonies belonging to PSH09 present common morphological characteristics: large colonies presenting robust erected or horizontal branches, rounded or flattened, with more or less pronounced verrucae that are uniform in shape and spacing. Nevertheless, the *corallum* macromorphology is not a diagnostic character in *Pocillopora* genus [e.g., Paz‐Garcia et al. (2015) or Gélin, Postaire, et al. (2017)]. It is present on all reef slopes and less frequently in lagoons, from surface to 40 m (HM, pers. obs.). Its three‐dimensional structure is an element of reef structuring, and its broad interbranch width provides habitats for a huge variety of species, making it a key species of coral reef ecosystems from the Indo‐Pacific and Red Sea. On a biological point of view, *P. eydouxi* morphospecies has been described as a broadcast spawner (Hirose, Kinzie, & Hidaka, 2001). Moreover, they evidenced that zooxanthellae were maternally inherited in oocytes, suggesting that larvae are autotrophic and intuitively meaning that their dispersal abilities are not limited by intrinsic resources as lecithotrophic larvae could be. Apart from these studies, focusing mainly on biology, ecology, and taxonomy, no one has investigated yet population genetic diversity in this species complex.

In front of such incomplete knowledge in *P. eydouxi*, it seems urgent to collect and reinforce data for this too long ignored scleractinian species complex despite its undeniable role in reef architecture and maintenance over the Indian Ocean, the Pacific Ocean, and the Red Sea. Thus, this study aimed to explore the cryptic genetic diversity within PSH09, and more precisely, within each SSH (SSH09a, b, c) using a combination of population genetics data (13 microsatellites) along with different kinds of clustering analyses, and so at different spatial scales (reef < island < ecoregion < province). The hierarchical sampling focused on three understudied provinces (from a genetic connectivity point of view): the Western Indian Ocean, the Tropical Southwestern Pacific, and Southeast Polynesia. This should allow refining species delimitation in this species complex, better estimating reef biodiversity and identifying the units on which connectivity should be assessed.

## 2 MATERIAL AND METHODS

### 2.1 Sampling

**Figure 1:**
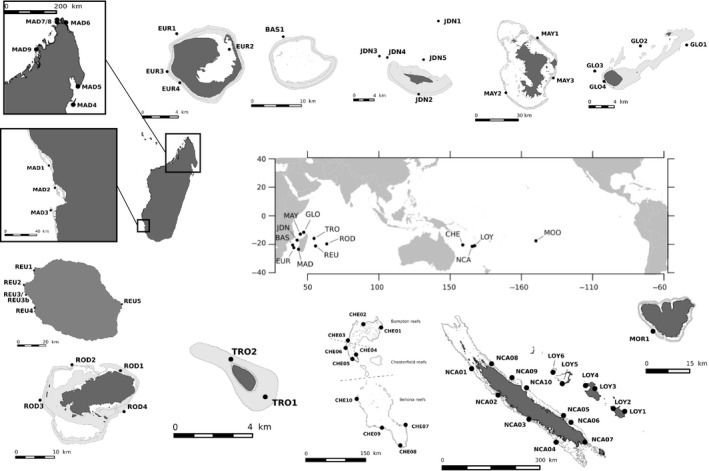
Sampling locations of *Pocillopora* colonies. Populations are numerically identified from the island code [GLO: Glorioso Islands, MAY: Mayotte, JDN: Juan de Nova Island; BAS: Bassas da India, EUR: Europa Island, MAD: Madagascar, REU: Reunion Island, ROD: Rodrigues Island, CHE: Chesterfield Islands, NCA: Grande Terre (New Caledonia), LOY: Loyalty Islands (New Caledonia), and MOR: Moorea (French Polynesia)]. Constructed using © OpenStreetMap contributors CC BY‐SA ( http://www.openstreetmap.org/copyright) for landmasses and UNEP‐WCMC, WorldFish Centre, WRI, TNC (2010). Global distribution of coral reefs, compiled from multiple sources including the Millennium Coral Reef Mapping Project. Version 1.3. Includes contributions from IMaRS‐USF and IRD (2005), IMaRS‐USF (2005) and Spalding et al. (2001). Cambridge (UK): UNEP World Conservation Monitoring Centre ( http://data.unep-wcmc.org/datasets/1) for coral reefs

In the aim of exploring the *Pocillopora* genus diversity and *in fine* studying population connectivity, colonies of *Pocillopora* genus were sampled [tip of branches + photographs except for Tromelin Island (Scattered Islands) and Polynesia] independently of their *corallum* macromorphology, as it is not a diagnostic character in *Pocillopora* genus. So species identification was realized molecularly *a posteriori* of sampling and *a priori* of data analyses (see below), leading to a subset of 2,507 colonies corresponding to PSH09 *sensu* Gélin, Postaire, et al. (2017). The sampling was achieved from April 2011 to October 2016, in three marine provinces extended over six ecoregions (Spalding et al., 2007): the Western Indian Ocean (WIO), the Tropical Southwestern Pacific (TSP), and the Southeast Polynesia (SEP). The sampling followed a hierarchical scheme with several islands within a province and several sites within an island (province > ecoregion > island > site; Figure 1, Table 1). It represented a total of 12 islands (included large islands: Madagascar and New Caledonia) and 65 sites. For a given site, colonies were usually sampled at the same depth (8–14 m), during one single dive, so that the range of sampling for each site did not exceed some hundreds of m² and the distance between two colonies within a site varied from few centimeters to few meters, depending on the density of *Pocillopora* colonies.

**Table 1:** Sampling of *Pocillopora eydouxi* (Primary Species Hypothesis PSH09 in Gélin, Postaire, et al. (2017)) colonies. For each site, are indicated the island, the ecoregion, and the province. The total number of sampled colonies (*N*_TOT_) and the number of sampled colonies per Secondary Species Hypothesis (SSH09a, SSH09b, and SSH09c) are indicated for each site

Province	Ecoregion	Island	Site	Site Name	Latitude	Longitude	*N*_TOT_	SSH09a	SSH09b	SSH09c
Western Indian Ocean	Western and northern Madagascar	Mayotte	MAY1	Grand Recif Nord‐Est	−12.62755	45.17750	45	45		
			MAY2	Passe Bateau	−12.97175	44.97857	35	31		4
			MAY3	Passe en S	−12.87910	45.27705	42	42		
		Glorioso Island	GLO1	Est	−11.5336	47.40126	31	31		
			GLO2	Nord	−11.53490	47.33545	29	29		
			GLO3	Sud‐Ouest	−11.57074	47.27066	45	45		
			GLO4	Sud	−11.58543	47.28376	19	19		
		Juan de Nova Island	JDN1	Biodiv 2	−16.95337	42.76011	58	57		1
			JDN2	Biodiv 6	−17.08177	42.72536	44	43		1
			JDN3	Biodiv 7	−17.01493	42.65645	48	48		
			JDN4	Nord‐Ouest	−17.01737	42.67027	31	31		
			JDN5	Nord	−17.02115	42.73402	29	29		
		Bassas da India	BAS1	Nord	−21.43418	39.65295	28	28		
		Europa	EUR1	Station météo	−22.33505	40.33439	60	60		
			EUR2	Pointe Est	−22.35081	40.38706	39	39		
			EUR3	Ouest	−22.37300	40.32483	9	9		
			EUR4	Sud‐Ouest	−22.38403	40.3375	11	10		1
		Madagascar (Tuléar)	MAD1	Anakao Nosy Vé	−23.15016	43.56768	48	48		
			MAD2	Ifaty Sud Passe	−23.40106	43.63468	49	48		1
			MAD3	Grand Récif de Tuléar	−23.65441	43.58764	68	68		
		Madagascar (north)	MAD4	Ste Marie	−17.119681	49.819741	10	10		
			MAD5	Cap Masoala	−16.01399	50.151709	30	30		
			MAD6	Baie de Diego	−12.22962	49.35554	41	39	1	1
			MAD7	Nosy Hara	−12.24219	49.01742	51	47	3	1
			MAD8	Nosy Hao	−12.09729	49.03414	28	28		
			MAD9	Radama	−14.11170	47.68078	67	62	5	
Mascarene Islands	Reunion Island	REU1	Le Port	−20.94338	55.27963	50	44	3	3
		REU2	Mahapany	−21.03401	55.21478	38	38		
		REU3	La Saline	−21.09812	55.22826	39	39		
		REU3b	Planch’ Alizé	−21.09778	55.23239	15	15		
		REU4	Saint‐Leu	−21.18029	55.28371	33	33		
		REU5	Sainte‐Rose	−21.15695	55.8362	47	47		
	Rodrigues	ROD1	Rivière Banane	−19.66726	63.46867	47	45		2
		ROD2	Mourouk (SEMPA)	−19.75397	63.46803	54	53		1
		ROD3	Boobies Island	−19.65073	63.40020	35	34		1
		ROD4	Iles auc Cocos	−19.73523	63.28396	47	47		
Cargados Carajos/Tromelin Island	Tromelin Island	TRO1	Pointe Sud	−15.90072	54.53346	16	16		
		TRO2	Nord‐Ouest	−15.88354	54.51773	16	16		
Tropical Southwestern Pacific	Chesterfield Islands	Bampton Reefs	CHE01	Ilot Reynard	−19.20365	158.93706	38		2	36
			CHE02	Bampton Nord	−19.11688	158.60000	39			39
			CHE03	Ilot Avon	−19.48349	158.25203	30			30
		Chesterfield Reefs	CHE04	Les Ilots du Mouillage	−19.80292	158.43595	38		6	32
			CHE05	Ilot du Passage	−19.90287	158.35330	61		5	56
			CHE06	Ilot de Sable	−19.65317	158.20108	50		2	48
		Bellona Reefs	CHE07	Récif Olry	−21.38758	159.55353	48			48
			CHE08	Récif de l'Anneau	−21.85115	159.43348	35		4	31
			CHE09	Récif du Milieu	−21.45272	159.02081	56		7	49
			CHE10	Bellona Nord‐Ouest	−20.80306	158.45104	53		5	48
	New Caledonia	Grande Terre	NCA01	Poum	−20.30244	163.88345	26		15	11
	NCA02		Voh	−21.0339	164.61812	42		34	8
	NCA03		Bourail	−21.70086	165.46918	31		10	21
	NCA04		Nouméa	−22.33536	166.2219	25		11	14
	NCA05		Port Bouquet	−21.59848	166.42591	54		4	50
	NCA06		Kuakué	−21.7857	166.63385	56		13	43
	NCA07		Goro	−22.33438	167.01815	48		19	29
	NCA08		Pouébo	−20.16931	164.43608	61		15	46
	NCA09		Hienghène	−20.55310	165.00238	38		13	25
	NCA10		Poindimié	−20.8337	165.40346	37		4	33
Loyalty Islands	LOY1	Maré	−21.40448	167.8007	10		3	7
	LOY2	Maré	−21.48428	168.11743	13			13
	LOY3	Lifou	−20.85568	167.29346	25		23	2
	LOY4	Lifou	−20.77063	167.03459	55		34	21
	LOY5	Ouvéa	−20.71891	166.39266	47		29	18
	LOY6	Beautemps‐Beaupré	−20.404	166.13475	49		43	6
Southeast Polynesia	Society Islands	Moorea	MOR1	Haapiti	−17.57569	−149.87803	10		10	
Total							2,507	1,403	323	781

### 2.2 DNA extraction, sequencing, and microsatellite genotyping

From the sampled colonies, DNA was extracted using DNeasy Blood & Tissue kit (Qiagen^™^). Genotyping was performed following the same protocol than in Gélin, Postaire, et al. (2017) and so using the same 13 microsatellite loci. PCR products were genotyped using an ABI 3730 genetic analyser (Applied Biosystems), and allelic sizes were determined with GeneMapper v.4.0 (Applied Biosystems) using an internal size standard (Genescan LIZ‐500, Applied Biosystems). Because colonies were sampled based on their *corallum* macromorphology, *P. eydouxi* lineage identity was verified *a priori* using assignment tests performed with structure (Pritchard, Stephens, & Donnelly, 2000). For this purpose, we combined all sampled colonies for this study and the 943 colonies from Gélin, Postaire, et al. (2017) that were assigned to the different PSHs as in Gélin, Postaire, et al. (2017). From that, a total of 2,507 colonies grouped together in PSH09 and constituted the final dataset.

### 2.3 MLG identification and assignment to the SSHs

To check for clonal propagation among the sampled colonies, identical multilocus genotypes (MLG) were identified using the R (R Development Core Team 2016) package *RClone* (Bailleul, Stoeckel, & Arnaud‐Haond, 2016).

Then, keeping one representative per MLG, we assigned the colonies to the three SSHs (SSH09a, SSH09b and SSH09c) previously identified in Gélin, Postaire, et al. (2017). To do so, we performed assignment tests on the 2,507 genotyped colonies, including the 257 colonies from PSH09 used in Gélin, Postaire, et al. (2017) and using structure (Pritchard et al., 2000) for *K *=* *3. Five chains with 2 × 10^6^ generation steps after a burn‐in of 2 × 10^5^ were run assuming admixture and correlated frequencies. The structure outputs were summarized with clumpp v.1.0 (Jakobsson & Rosenberg, 2007) and drawn with distruct v.1.1 (Rosenberg, 2004).

The colonies showing an assignment probability for each of the five runs >0.75 to a given SSH were assigned to this SSH (practically identified by a unique color on Figures). Colonies that did not show an assignment >0.75 for a given SSH were considered admixed, that is, assigned to more than one SSH (due to hybridization or shared ancestry or bad assignment due to missing data). For each admixed colony, it was assigned to the SSHs presenting an assignment probability >0.1 (a probability < 0.1 was considered as noise). Moreover, we used newhybrids v.1.1 (Anderson & Thompson, 2002), running 5 × 10^5^ iterations after a burn‐in period of 5 × 10^4^, to detect whether the admixed colonies assigned to two SSHs found in structure could be considered as hybrids. newhybrids calculates the posterior probability that sampled colonies fall into each of a set of hybrid categories (Parent 1, Parent 2, F1, F2, backcross to Parent 1, backcross to Parent 2). Once identified, the admixed colonies were not considered in the subsequent analyses.

The genotypic linkage disequilibrium was assessed with fstat v.2.9.3 both on the whole dataset and for each SSH separately (Goudet, 2001), and pairwise differentiation among SSHs was estimated using the *F*_ST_ (Weir & Cockerham, 1984) with arlequin v.3.5 (Schneider, Roessli, & Excoffier, 2000) and *D*_est_ using the R package *DEMEtics* (Gerlach, Jueterbock, Kraemer, Deppermann, & Harmand, 2010). For each SSH, the allelic frequency distributions were plotted, and the number of alleles (*Na*) and the number of private alleles (*Np*) were estimated using fstat v.2.9.3 (Goudet, 2001). Then, considering the whole dataset, the global *F*_ST_ (Weir & Cockerham, 1984) for each locus, was assessed with fstat v.2.9.3.

### 2.4 Structuring analyses within each SSH

Further analyses were performed on each SSH separately. First, to determine the most likely number of genetically homogenous groups (*K*) within each SSH, a Bayesian analysis was performed using Structure v.2.3.4 (Pritchard et al., 2000) with the same conditions as described above. This analysis assumes that, within a set of samples, there are *K* genetic groups and colonies are assigned to each putative genetic groups under Hardy–Weinberg equilibrium (HWE) and minimized linkage disequilibrium (LD). To allow comparing the different outputs from the different types of analyses (see below), the colonies were assigned to clusters following the same rule than above (replacing SSH by cluster, and one color per cluster) and the same rule was applied to constitute the dataset (i.e., removing admixed colonies) for further analyses. As structure demands strong assumptions on the genetic groups identified, we performed in parallel a discriminant analysis of principal components (DAPC; Jombart, Devillard, & Balloux, 2010) in order to test whether the structuring observed with Structure v.2.3.4 could be retrieved with DAPC, as this latter analysis does not make any assumption about HWE or LD (it transforms genotypes using PCA as a prior step to a discriminant analysis). DAPC was applied using the *adegenet* package (Jombart, 2008) for R (R Development Core Team 2016). Then to evaluate the congruency between structure and DAPC for a given *K*, the number of colonies that were assigned differently between both methods was estimated.

In a hierarchical approach, these analyses were repeated on each cluster found in each SSH separately. Commonly, using structure and DAPC, when the finest level of structuring is reached, adding a supplementary cluster leads to inconclusive assignments with colonies assigned to several clusters in the same proportions. Here, some colonies kept ongoing assigned to clusters when *K* increased above the most likely *K*, either using structure or DAPC. Moreover, because the methods traditionally used to detect the most likely number of genetic groups within a dataset [Pr(X|*K*) (Pritchard et al., 2000), Δ*K* method (Evanno, Regnaut, & Goudet, 2005), the deviance information criterion (DIC) (Gao, Bryc, & Bustamante, 2011), the Bayesian information criterion (BIC) (Jombart et al., 2010), and the thermodynamics integration (TI) (Verity & Nichols, 2016)] might provide different outputs, and because they are purely mathematical, they might not reflect the biological truth. So to exclude mathematical artefacts, we chose to consider the highest number of genetic groups corresponding to the clustering and the individual assignments that were retrieved by all the four possible combinations [(structure *vs*. DAPC) × (all colonies of a given SSH *vs*. each cluster within this SSH separately)].

Considering the clusters finally kept within each SSH, the pairwise differentiation among clusters within each SSH was estimated using the *F*_ST_ (Weir & Cockerham, 1984) with arlequin v.3.5 (Schneider et al., 2000) and *D*_est_ using the R package *DEMEtics* (Gerlach et al., 2010). Then, for each cluster, the allelic frequencies spectrum for each locus were plotted, and considering each SSH, the global *F*_ST_ (Weir & Cockerham, 1984) for each locus was calculated using fstat v.2.9.3 (Goudet, 2001). Additionally, minimum spanning trees based on the shared alleles distance (DAS) were performed with edenetwork (Kivelä, Arnaud‐Haond, & Saramäki, 2015) and drawn with colonies colored according to the clusters found in the assignment analyses. Moreover, we used newhybrids v.1.1 (Anderson & Thompson, 2002) between pairs of clusters within each SSH to detect whether the admixed colonies found in structure could be considered as hybrids (same conditions as above). Finally, a hierarchical analysis of molecular variance (AMOVA) was performed using arlequin v.3.5 (Schneider et al., 2000) considering the whole dataset with SSH as group and clusters within SSH as populations.

## 3 RESULTS

### 3.1 MLG identification and assignment to the SSHs

**Figure 2:**
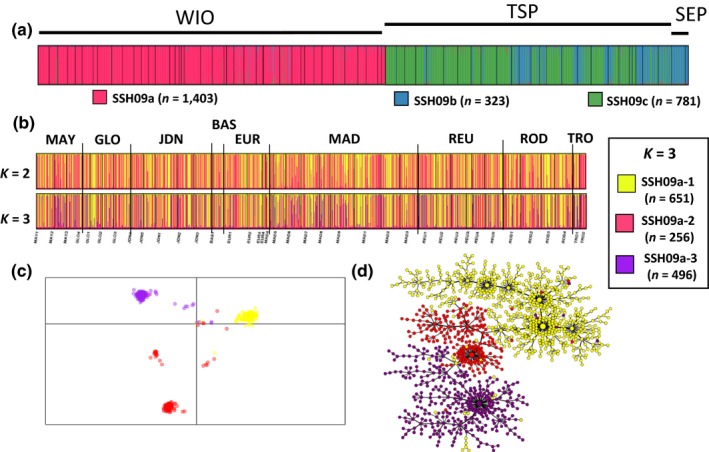
SSH09a clusters. (a) Results of the assignment tests for PSH09 at *K *=* *3 and (b) the structure plots for *K *=* *2 and *K *=* *3 are presented. (c) Results of the DAPC assignment for *K *=* *3 and (d) the minimum spanning tree, both colored according to the three clusters identified by structure

Among the 2,507 *Pocillopora* colonies assigned to PSH09 (Gélin, Postaire, et al. (2017)), each represented a unique MLG. First, assigning the colonies to the three SSHs, all the colonies from the TSP and the SEP (*n* = 1,075) were assigned to both SSH09b and SSH09c (none to SSH09a), while almost all the colonies from the WIO (*n* = 1,430) were assigned to SSH09a (*n* = 1,403), except 12 and 15 that were assigned to SSH09b and SSH09c, respectively (Table 1; Figure 2). Interestingly, looking at the site REU1 (*n* = 50) in Reunion Island, 44 colonies were assigned to SSH09a, three to SSH09b, and three to SSH09c. Noteworthy, all the colonies showed a probability of assignment to each SSH > 0.75, except six colonies (0.2%) that were found to be admixed between SSHs: one colony from New Caledonia (LOY6) was admixed between SSH09b and SSH09c, and five from North Madagascar were admixed: one (MAD8) between SSH09a and SSH09c, two colonies (MAD5 and MAD6) between SSH09a and SSH09c, and two (MAD6 and MAD7) between all the SSHs. Nevertheless, the multilocus genotypes of all these admixed colonies presented missing data (37% in average) and might just reflect a bad assignment. Additionally, newhybrids detected only 18 hybrids over 2,507 colonies (0.7%), and all were F2 hybrids. Moreover, a high proportion of colonies assigned to one of the three SSHs identified with structure (i.e., assignment probability > 0.75) were assigned as pure lineages with newhybrids (i.e., 99.3, 98.1 and 98.8% for SSH09a, SSH09b, and SSH09c, respectively).

Inter‐SSHs genetic differentiation varied from 0.158*** to 0.248*** for the *F*_ST_ estimates and from 0.376*** to 0.430*** for the *D*_est_ ones (Table 2). Then, considering the whole dataset irrespective to SSHs, 10 tests of genotypic disequilibrium were significant over 78, indicating 13% of genotypic disequilibrium among the 13 loci after Bonferroni correction, while, considering each SSH separately, none of the 78 tests revealed disequilibrium within both SSH09a and SSH09c and 10 of 78 within SSH09b.

**Table 2:** Genetic differentiation among the three identified Secondary Species Hypotheses estimated with Weir and Cockerham's *F*_ST_ (lower diagonal; Weir & Cockerham, 1984) and with Jost's *D*_est_ (upper diagonal; Jost, 2008). In parentheses, is indicated the number of colonies. *** *P* < 0.001

	SSH09a	SSH09b	SSH09c
SSH09a (1,403)	–	0.402***	0.438***
SSH09b (323)	0.158***	–	0.376***
SSH09c (781)	0.248***	0.225***	–

The mean number of alleles (± standard error) per locus was high, varying from 13.00 ± 0.87 for SSH09b to 14.54 ± 1.27 for SSH09c (Appendix S1). For each SSH, between 11 and 14% of private alleles were identified, the mean number of private alleles per locus varying from 1.46 ± 0.33 for SSH09c to 1.85 ± 0.41 for SSH09b (Appendix S1). The allele frequency spectrum for each locus and for each SSH as well as the *F*_ST_ per locus considering the whole dataset (Appendix S2) showed some high values from 0.009* to 0.769*** for Pd3‐008 and a high variance (mean *F*_ST_ ± *SE *=* *0.188 ± 0.058). In addition, loci Pd11 and Pd13 showed a weaker amplification rate on colonies from SSH09a than from SSH09b and SSH09c (data not shown).

Overall, this indicates a high genetic differentiation among the three SSHs, indicating restricted gene flow between these SSHs. Thus, from now, we will consider these three SSHs as independent genetic lineages and will perform the subsequent analyses on each SSH separately (*N*_SSH09a_ **=* *1,403; *N*_SSH09b_ **=* *323; *N*_SSH09c_ **=* *781; Table 1).

### 3.2 Structuring analyses within each SSH

#### 3.2.1 SSH09a

Considering all the colonies assigned to SSH09a (exclusively located in the WIO; *n *=* *1,403), DAPC showed nearly similar individual assignments than structure for both *K *=* *2 and *K *=* *3: for *K *=* *2, we observed two clusters, SSH09a‐1 and SSH09a‐2, while for *K *=* *3, SSH09a‐2 was further divided into two clusters (SSH09a‐2 and SSH09a‐3, Figure 2). To explore in depth the partitioning, we re‐analyzed alone the first cluster (SSH09a‐1) found for *K *=* *2: it did not separate anymore using structure nor DAPC. Conversely, the second cluster (SSH09a‐2) when re‐analyzed alone did split in three distinct subclusters with structure while with DAPC, two of these subclusters were overlapping. Then, reconsidering all the colonies and now *K *=* *4, structure did find four clusters, but they did not correspond to the ones found using DAPC, nor to the ones found when treating the two‐first clusters independently (in this case, SSH09a‐1 and SSH09a‐2 both split into two).

Because our choice criterion was the congruency of the different methods [(structure *vs*. DAPC) × (all colonies *vs*. substructuring)], we finally considered that all the colonies from SSH09a could be divided into three distinct clusters (SSH09a‐1, SSH09a‐2 and SSH09a‐3; Figure 2). At *K *=* *3 in structure, each colony was assigned identically among the five runs. Then, 1,310 colonies (94%) were assigned to a unique cluster (*N*_SSH09a‐1_ **=* *600, *N*_SSH09a‐2_ **=* *237, and *N*_SSH09a‐3_ **=* *473), and 93 colonies (6%) were admixed in several clusters: 29 colonies (2%) between SSH09a‐1 and SSH09a‐2, 10 colonies (0.7%) between SSH09a‐1 and SSH09a‐3, 29 colonies (2%) between SSH09a‐2 and SSH09a‐3, and 25 colonies (1.7%) among the three clusters. Nevertheless, only 12 of these 93 colonies presented no missing data, suggesting that the other admixed colonies (21% of missing data in average) might be the result of bad assignment. Comparing both methods, only 4.7% of the colonies were assigned differently. The minimum spanning tree retrieved the three clusters (Figure 2d). Surprisingly, these latter were found nearly evenly distributed in all the sampling sites with no apparent geographical pattern, cluster SSH09a‐1 being the most represented (46%; Figure 2). Pairwise *F*_ST_ estimates among the three clusters were of the same order, between 0.125*** and 0.150*** (Table 3). Comparatively, *D*_est_ values appeared lower, comprised between 0.085*** and 0.092*** (Table 3) but all significantly different from zero. Global *F*_ST_ per locus ranged from 0.000 ^ns^ to 0.772*** for PV7 (mean *F*_ST_ ± *SE *=* *0.061 ± 0.062; Appendix S3).

**Table 3:** Genetic differentiation between the three identified clusters within SSH09a estimated with Weir and Cockerham's *F*_ST_ (lower diagonal; Weir & Cockerham, 1984) and with Jost's *D*_est_ (upper diagonal; Jost, 2008). In parentheses, is indicated the number of colonies . *** *P*<0.001

	SSH09a‐1	SSH09a‐2	SSH09a‐3
SSH09a‐1 (651)	–	0.090***	0.092***
SSH09a‐2 (256)	0.125***	–	0.085***
SSH09a‐3 (496)	0.140***	0.150***	–

#### 3.2.2 SSH09b

**Figure 3:**
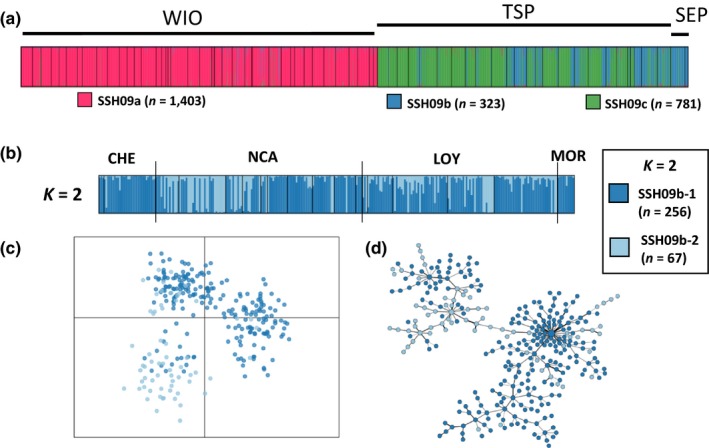
SSH09b clusters. (a) Results of the assignment tests for PSH09 at *K *=* *3 and (b) the structure plots for *K *=* *2 are presented. (c) Results of the DAPC assignment for *K *=* *2 and (d) the minimum spanning tree, both colored according to both clusters identified by structure

Concerning SSH09b (*n *=* *323; nearly exclusively found in the South Pacific), for *K *=* *2, colonies from the WIO, Chesterfield Islands, and French Polynesia were assigned in the first cluster (SSH09b‐1; Figure 3b), while colonies from New Caledonia in both clusters (SSH09b‐1 and SSH09b‐2) with no apparent geographical pattern. Moreover, colonies of each cluster were not evenly spread across sites. For *K *=* *3 and *K *=* *4, individual assignments were not congruent between structure and DAPC (data not shown), even when analyzing both clusters separately.

Thus, we considered that SSH09b split into two clusters, [SSH09b‐1 (*n* = 256) and SSH09b‐2 (*n* = 67); Figure 3]. For *K *=* *2 in structure, each colony was assigned identically among the five runs. Then, 307 colonies (95%) were assigned to a unique cluster (*N*_SSH09b‐1_ **=* *244 and *N*_SSH09b‐2_ **=* *63) and 16 colonies (5%) were admixed between SSH09b‐1 and SSH09b‐2. Nevertheless, only three colonies presented no missing data, suggesting that the 13 other admixed colonies (23% of missing data in average) might be the result of bad assignment. Only 2.9% of the colonies were assigned differently between both methods. The minimum spanning tree retrieved the two clusters (Figure 3d), which were highly differentiated from each other (*F*_ST_ **=* *0.131*** and *D*_est_ **=* *0.259***). Global *F*_ST_ per locus ranged from 0.001 ^ns^ to 0.283*** for Poc40 (mean *F*_ST_ ± *SE *=* *0.104 ± 0.029; Appendix S4).

#### 3.2.3 SSH09c

**Figure 4:**
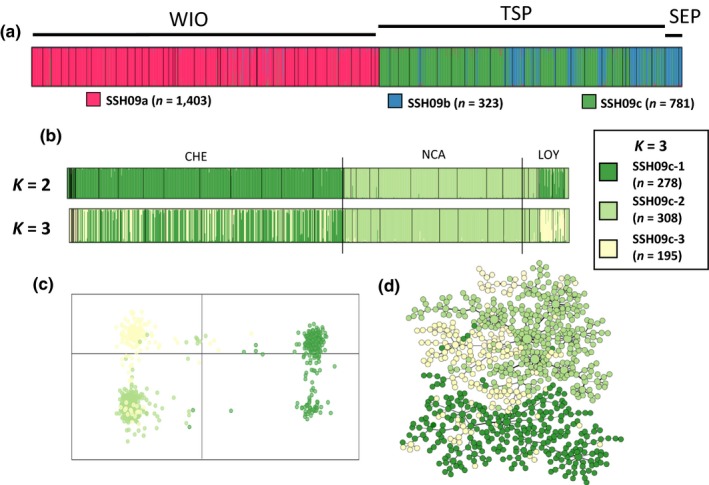
SSH09c clusters. (a) Results of the assignment tests for PSH09 at *K *=* *3 and (b) the structure plots for *K *=* *2 and *K *=* *3 are presented. (c) Results of the DAPC assignment for *K *=* *3 and (d) the minimum spanning tree, both colored according to the three clusters identified by structure

Concerning SSH09c (*n *=* *781), for *K *=* *2, the structuring pattern nearly corresponded to a geographical pattern. Indeed, on one hand, colonies from Chesterfield Islands together with those of LOY4, LOY5 (Loyalty Islands) and the 15 colonies from the WIO were grouped in a first cluster (SSH09c‐1; Figure 4b), and on the other hand, all the other colonies from New Caledonia (Grande Terre and Loyalty Islands) composed the second cluster (SSH09c‐2; Figure 4b). For *K *=* *3, SSH09c‐1 further split into two clusters, the colonies from Loyalty Islands all appearing differentiated in a third cluster (SSH09c‐3) while colonies from Chesterfield Islands segregated in both SSH09c‐1 and SSH09c‐3. Using DAPC, the same partitioning was observed either for *K *=* *2 or *K *=* *3 (Figure 4b).

Thus, following our choice criterion, three clusters were identified. At *K *=* *3 in structure, each colony was assigned identically among the five runs. Then, 756 colonies (97%) were assigned to a unique cluster (*N*_SSH09c‐1_ **=* *273, *N*_SSH09c‐2_ **=* *302, and *N*_SSH09c‐3_ **=* *181), and 25 colonies (3%) were admixed in several clusters: two colonies between SSH09c‐1 and SSH09c‐2, 11 colonies between SSH09c‐1 and SSH09c‐3, nine colonies between SSH09c‐2 and SSH09c‐3, and three colonies among the three clusters. Nevertheless, only four colonies presented no missing data, suggesting that the other admixed colonies (19% of missing data in average) might be the result of bad assignment. Only 1.9% of the colonies were assigned differently among the two methods. The three clusters were retrieved in the minimum spanning tree (Figure 4d), and genetic differentiation among them was high, either considering *F*_ST_ (from 0.16*** to 0.34***) or *D*_est_ (from 0.09*** to 0.31***) estimates (Table 4). Global *F*_ST_ per locus ranged from 0.001 ^ns^ to 0.737*** for Pd4 (mean *F*_ST_ ± *SE *=* *0.097 ± 0.063; Appendix S5).

**Table 4:** Genetic differentiation between the three identified clusters within SSH09c estimated with Weir and Cockerham's *F*_ST_ (lower diagonal; Weir & Cockerham, 1984) and with Jost's *D*_est_ (upper diagonal; Jost, 2008). In parentheses, is indicated the number of colonies. *** *P* < 0.001

	SSH09c‐1	SSH09c‐2	SSH09c‐3
SSH09c‐1 (278)	–	0.310***	0.087***
SSH09c‐2 (308)	0.339***	–	0.219***
SSH09c‐3 (195)	0.163***	0.221***	–

Additionally, within each SSH, only F2 hybrids were detected using newhybrids between pairs of clusters. Nevertheless, colonies assigned to a unique cluster with structure (assignment probability > 0.75) were mainly assigned in pure lineages with newhybrids (i.e., 97, 89.4 and 98.7% in average among the clusters of SSH09a, SSH09b, and SSH09c, respectively).

Finally, considering this hierarchical clustering of the whole dataset (three SSHs splitting in several clusters), the AMOVA revealed that nearly 15% of the overall genetic variation was explained by the partition into three SSHs and 12% by the partition into clusters within SSH (Appendix S6).

## 4 DISCUSSION

### 4.1 Russian dolls

The present study focuses on the genetic variability among colonies from the Primary Species Hypothesis PSH09 found in Gélin, Postaire, et al. (2017). PSH09 corresponds to two ORF and four Dloop haplotypes and was attributed *P. eydouxi* name. In this previous analysis, based on microsatellite loci, Gélin, Postaire, et al. (2017) identified three genetically distinct clusters within PSH09, two of which were found in sympatry, leading to the recognition of three Secondary Species Hypotheses within PSH09 (SSH09a, b, and c). Here, using microsatellite data and assignment tests on a much larger sampling, we further revealed that each of them was found in sympatry with one or the other, with the three being found on a single reef in the WIO (Reunion Island, REU1). Furthermore, according to our choice criterion to determine the number of genetic groups *K*, we even found that each of these SSHs was partitioned into two to three additional clusters, which were also all found in sympatry at least at the island scale within the distribution range of their respective SSH, with very rare hybrids (only F2 and none F1). Noteworthy, these clusters were as much genetically differentiated from each other as SSHs were, or as SSHs belonging to different PSHs were [see Appendix S13 in Gélin, Postaire, et al. (2017)]. To summarize, when dissecting the genetic variability of PSH09, we revealed several nested hierarchical levels (PSH > SSH > cluster), each level hiding highly differentiated genetic groups as in Russian dolls, not in agreement with geography for most of them.

This pattern of interspersed genetic clusters among populations, revealed using structure assignments and microsatellite data, has already been reported in the literature. In the coral *Seriatopora hystrix* in Japan, Nakajima et al. (2017) found three interspersed genetic clusters in sympatry among the sampled sites corresponding to three distinct genetic lineages identified by mitochondrial DNA. Aside from a mix of mitochondrial lineages, the existence of ecological gradients has been proposed to explain a mix of clusters at the site level. As an illustration, Van Oppen, Bongaerts, Underwood, Peplow, and Cooper (2011)found that colonies of *S. hystrix* in North Australia were assigned in genetic clusters that corresponded to depth. Moreover, they evidenced vertical migration between shallow and deep habitats in each site explaining the mix of clusters found for some particular depths. Serrano et al. (2016) also revealed a pattern of depth structuring for *Porites astreoides* from Florida. Zayasu et al. (2016), studying *Acropora tenuis* in southwestern Japan, hypothesized the existence of two source populations (two genetic clusters) in both farthest sites of the sampling area and a mix of both clusters in the middle of the sampling area that should be linked to migration from extreme sites to the intermediate ones. A similar pattern was observed in *P. damicornis* β (Gélin, Fauvelot, Mehn, Bureau, Rouzé & Magalon, 2017) in Reunion Island with both clusters mixed in one intermediate site, evoking a geographic barrier or an asynchronous spawning of the most remote populations. In the Caribbean coral *Orbicella faveolata*, Porto‐Hannes et al. (2015) found two genetic clusters in sympatry at the reef scale, mainly on two sites from Belize. They suggested that those sites would have been more recently colonized than other sites because the sampled colonies were smaller than the colonies from the other sites, reflecting a reduction in larval production due to mortality that could favor local recruitment. Jia et al. (2016) found six genetic clusters interspersed among seven populations of the sandy shrub *Salix psammophila*, but they did not propose any hypothesis to explain this pattern. Finally, with the growing body of population genetics studies, these patterns of mixed clusters among populations are increasingly revealed without understanding clearly the underlying processes.

### 4.2 Is this the real life? Is this just fantasy?

Here, we used microsatellite genetic data coupled with different methods to refine lineage boundaries within the *P. eydouxi* complex (PSH09; Gélin, Postaire, et al. (2017)). Thus, despite the fact that microsatellites are not commonly used in species delimitation but rather in population genetics, their high polymorphism can help in refining boundaries between closely related species (e.g., Dawson et al., 2010). Although structure and DAPC differ in their *a priori* hypotheses, we observed very few differences (3.6% over the whole dataset) between the outputs of these two methods, highlighting their congruency and comforting us that the observed clusters were independent of the method. The main problem when using clustering methods is to determine the true number of genetically differentiated clusters, *K*, present in the dataset, subject of debate over the last decade. Nevertheless, all the estimators of *K* traditionally used can all give different values for a given dataset, and not a single estimator can provide a true value of *K*, as suggested by Jombart et al. (2010). Recently, Verity and Nichols (2016) suggested that *K* should be viewed as a flexible parameter that describes just one point on a continuously varying scale of population structure. Therefore, comparing the outputs of the different analyses and keeping the highest value of *K* that gave congruent results among analyses seemed to be a good compromise to estimate the best number of genetic groups.

Overall in the whole sampling (2,507 colonies), we did not find at least two colonies sharing the same MLG, suggesting that the analyzed colonies do not exhibit clonal propagation, in accordance with their massive morphology and their stout branches, limiting fragmentation. So the genotypic linkage observed in the whole dataset was not due to the presence of repeated MLGs (clones), but to some particular alleles that were preferentially associated within SSH, as well as in SSH09b considering the non‐random association of alleles within each cluster. Thus, (1) the number of private alleles found in each SSH and in each cluster within SSH along with (2) the variation of allele frequencies among SSHs and also among clusters within SSH (high values of global *F*_ST_ per locus) both played a role in the genetic differentiation observed among SSHs and clusters.

Finally, rather than structured populations within a single species, these three SSHs, and even the eight clusters, likely represent distinct genetic lineages, though incompletely sorted for the ORF gene, engaged in a speciation process (half‐way in the “gray zone” between two populations highly differentiated and two sister species) or real species following the unified concept of De Queiroz (2007). In this way, Johnston et al. (2017) highlighted a genetic distinction between *P. eydouxi* and *P. meandrina* (which are not diagnosable using the ORF mitochondrial marker), suggesting that both might be recognizable using nuclear DNA. Thus, the SSHs (or even clusters) we observed might be the reflection of the presence of these two genetic lineages among our samples distinguished by the set of microsatellites used. The issue is now to understand in our case which hierarchical level (SSH, cluster or even below) corresponds to the species one. An extended integrative approach (microstructure, microenvironment ecology, symbionts, and genomics) is needed to fully conclude where to put the boundaries between species, whether these three SSHs actually correspond to three distinct species or whether each SSH would represent a complex of species, each cluster being a single species (or a complex of, more or less cryptic) with its own distribution area.

### 4.3 Origin of divergence

Even if we cannot fully conclude regarding the status of each of these SSHs and clusters, we can discuss the possible processes that lead to the appearance of such genetically divergent lineages, even if the use of microsatellites does not allow estimating the time of divergence among lineages.

Starting with the first hierarchical level, the three SSHs may re‐present ancestral lineages that were isolated in the past and evolved separately for a period of time long enough for reproductive barriers to emerge. The fact that SSH09a is restricted to WIO while SSH09b and SSH09c are almost exclusively found in the South Pacific Ocean strongly suggests the role of the Indo‐Pacific Barrier, a widely recognized partition (based on faunal distributions) that separates the Pacific and Indian Ocean provinces (Briggs, 1974), in generating the genetic divergences among SSHs. With the onset of Pleistocene glaciation cycles about 3 million years ago (Mya), global sea levels have fluctuated with maximum amplitudes of up to 140 m (Lambeck, Esat, & Potter, 2002). The sea level reached 120 m below present‐day level twice over the last two glacial periods, with the last one, occurring ca. 17–18,000 years ago, largely exposing Sunda and Sahul Shelves and restricting water exchanges between Indian Ocean and Pacific Ocean (Bard et al., 1996; Voris, 2000). As a consequence, the opportunity for genetic exchange for marine organisms between the two oceans strongly decreased and populations evolved independently on both sides of this semi‐permeable barrier, providing an occasion for population differentiation and incipient speciation to occur. Meanwhile, these sea‐level regressions profoundly affected the distribution of shallow‐water reef habitats (Lambeck & Chappell, 2001), generating population size reductions in reef‐associated organisms, which may have caused local extinction of some species (Fauvelot, Bernardi, & Planes, 2003). Although the magnitude and timing of relative sea‐level stands vary across the Indo‐Pacific region (Woodroffe & Horton, 2005), during low sea‐level periods, viable populations survived in coral reef refuges that were isolated from each other (Pellissier et al., 2014). Once sea levels rose again (to reach present‐day level), surviving populations, by then forming differentiated lineages, re‐expanded to reach their current geographic distributions (Fauvelot et al., 2003).

Then, several nonexclusive hypotheses may explain the nested partitioning within each SSH (i.e., the presence of differentiated clusters in sympatry at the island scale):

1. In all our provinces, genetically divergent clusters within SSH might be the result of a systematic sampling of sink populations without source populations, assuming that (a) these latter are assigned in one unique cluster each, (b) colonies belonging to one cluster do not reproduce with colonies from other clusters, implying some reproductive barriers among clusters (otherwise, we should observe more genetically homogeneous populations or hybrids), and (c) the source populations provide in individuals each sink population equally.
2. Alternatively, genetically divergent clusters within SSH may represent past clonal lineages. Reproductively isolated clones (pre‐ or post‐zygotic barriers impeding reproduction with closely related individuals) would have spread and diversified resulting in the different clusters observed. Nevertheless, as we did not detect clonal reproduction in our sampling, it seems unlikely, although clonal propagation has been evidenced in the TEP (Baums et al., 2014; Pinzón, Reyes‐Bonilla, Baums, & LaJeunesse, 2012) for ORF type 1 colonies [*sensu* Pinzón et al. (2012)]. If so, clonal reproduction may represent an ancestral (or re‐acquired) character in the TEP (east margin of *Pocillopora* distribution area), which would have been lost in other localities. However, the colonies studied (Baums et al., 2014; Pinzón et al., 2012) were described as *P. damicornis*‐like colonies, a morph that presents thin branches susceptible to break, favoring fragmentation.
3. Populations experiencing a reduction in gene flow (such as in allopatric speciation processes) could exhibit heterogeneity in genetic differentiation along the genome (Tine et al., 2014). This heterogeneity could lead to consider two populations as two distinct species when looking at the highly differentiated zones of the genome. To date, we ignore where the microsatellite loci we used are located [except PV7 which is in the Internal Transcribed Spacer 1 (HM pers. com. and Magalon, Samadi, Richard, Adjeroud, & Veuille, 2004)] and whether they could be located in highly differentiated zones of the genome. Additionally, the presence of *F*_ST_ outliers in divergence hitchhiking regions can largely influence the presence of sub‐structuring (reviewed in Via, 2012) and be a sign of adaptive divergence (Kulmuni & Westram, 2017). In our case, we highlighted some loci showing high values of *F*_ST_ for the different levels: PSH09 is split into three SSHs mainly because of Pd3‐008 (*F*_ST_ **=* *0.769***), SSH09a into three clusters mainly because of PV7 (*F*_ST_ **=* *0.772***) and SSH09c into three mainly because of Pd4 (*F*_ST_ **=* *0.734***). Nevertheless, each of these loci does not show any diagnostic allele, and taken separately from the others, it does not allow to retrieve the partitioning observed that likely results from the evolutionary history of all loci.
4. Hybridization could also explain the unexpected clustering pattern, each cluster being the result of different mixes between two sufficiently different genetic entities, either not sampled or ancestral. Indeed, hybridization is not uncommon in corals (e.g., Flot et al., 2011; Frade et al., 2010; Isomura, Iwao, & Fukami, 2013; Márquez, Van Oppen, Willis, Reyes, & Miller, 2002; Thomas et al., 2014; Vollmer & Palumbi, 2002). As an example, *Acropora prolifera* has been evidenced to be a hybrid (and not a hybrid species, see Willis, Van Oppen, Miller, Vollmer, & Ayre, 2006) between *A. cervicornis* and *A. palmata* (Van Oppen, Willis, Vugt, & Miller, 2000). Likewise, Combosch and Vollmer (2015) revealed hybridization between *Pocillopora* type 1 and type 3 using RADseq. Moreover, each time hybridization has been demonstrated in *Pocillopora* corals (Combosch, Guzman, Schuhmacher, & Vollmer, 2008; Combosch & Vollmer, 2015; Pinzón & LaJeunesse, 2011), it seemed dominated by type 1 maternal lineages since all hybrid colonies exhibited mitochondrial type 1 haplotypes (Combosch & Vollmer, 2015). Frequent events of hybridization could comfort Veron's suggestion about metaspecies (or syngameon) existence in corals (Veron, 1995). Whichever the causes, PSH09 (and even the whole *Pocillopora* genus) may represent a metaspecies with some hybrids between entities (such as the colonies that were assigned to two different SSHs or clusters herein). Nevertheless, a recent study resolving the phylogenetic relationships among seven species of *Pocillopora* using RAD‐seq did not provide any proof for hybridization among *P. eydouxi* and other *Pocillopora* species which were all found to be reciprocally monophyletic, although possible introgressive hybridization may have occurred between the most recently derived sister species *P. damicornis* and *P. acuta* (Johnston et al., 2017).
5. The observed clusters within SSH could be the result of habitat selection by individuals that could have specialized in different habitat types. Habitat selection has already been highlighted as a speciation factor. Indeed, for European anchovy populations, the habitat type (marine vs. coastal) accounts for most of the genetic structuring (Le Moan, Gagnaire, & Bonhomme, 2016): the genomic approach strongly supported a model of ecotypic divergence shaped by recent differential gene flow after a period of complete isolation (Le Moan et al., 2016). For corals, microhabitat selection could be the result of variability in light, current exposure, salinity, food availability, and/or temperature. Summarizing different habitat particularities, depth could be a factor of population structuring according to habitat type (e.g., *S. hystrix* in Australia; Van Oppen et al., 2011). However, in our case, colonies within each sampling site were systematically collected during a single dive, usually at the same depth (8–14 m) along a linear transect, and the clusters were not segregated in space within a site. Moreover, in Madagascar, while the colonies from two sites were found at different depths (MAD7, a reef flat between 1 and 2 m and MAD9, a pinnacle between 13 and 26 m), no genetic differentiation was found related to depth. Likewise, previous studies failed to detect a link between depth and genetic groups in the *Seriatopora* genus (Flot et al., 2008; Nakajima et al., 2017). All in all, while we cannot fully reject this hypothesis, depth does not appear an explanatory factor of genetic differentiation in our case, but microhabitat might be, considering fine‐scale variations of both abiotic and biotic factors that could be indiscernible to the human eye. Particularly, coral larvae are attracted to the substratum by sensory receptors (e.g., Tran & Hadfield, 2012) and are notably sensitive to biofilms produced by crustose coralline algae (e.g., Morse, Hooker, Morse, & Jensen, 1988). Therefore, the affinity for different kinds of coralline algae may play a role in micro‐habitat selection, as revealed in the pea aphid for which chemosensory gene families are determinant in host plant specialization (Smadja et al., 2012).
6. Finally, host–symbiont associations could play a role in the structuring pattern observed in this study. The evolution of coral hosts and their endosymbionts remains unclear. As an illustration, studying 69 genera (20 families) of octocorals and their endosymbionts, Van Oppen, Mieog, Sánchez, and Fabricius (2005) revealed that the symbiotic associations (at the level of phylogenetic clades) are not easily explained by taxonomic affiliation of the hosts. Nevertheless, host–symbiont association could be, on the contrary, more specific at the genus level. Indeed, Pinzón and LaJeunesse (2011) found some exclusive associations between subclades of *Symbiodinium* Clade C and ORF type of *Pocillopora* [e.g., C1b‐c and D were exclusively found in association with *Pocillopora* type 1 (PSH09 herein)]. The host–symbiont association should be further studied considering the clusters within the three SSHs to investigate the existence of specific associations at the different levels of genetic structuring in this host.

To date, we cannot favor one or another hypothesis and more investigations are needed to fully conclude regarding the origin of the genetically divergent clusters observed within PSH09. Several hypotheses imply an ancestral reduction in gene flow that created reproductive barriers or genome incompatibilities among the different clusters for each SSH, which contemporary gene flows have not homogenized yet.

## 5 CONCLUSION AND PERSPECTIVES

Examining the population structure of the *Pocillopora eydouxi* species hypothesis (PSH09; Gélin, Postaire, et al. 2017) revealed a nested partitioning of the different SSHs, obliging to think about the unit on which connectivity should be assessed. Whatever the causes, facing to this over‐partitioning of our dataset, the matter is not how to estimate connectivity but on what. As each SSH is a mix of several genetically differentiated clusters found in sympatry, we prefer considering the eight different clusters as our reference unit to assess genetic differentiation among populations.

Several hypotheses have been proposed to explain the observed pattern, but more investigations are needed to understand the structuring pattern of this species complex. Knowing more about *Pocillopora* genome seems now to be a fundamental key to improve the understanding of its history of divergence and would offer clues to favor one or another exposed hypothesis.

## CONFLICT OF INTEREST

None declared.

## DATA ACCESSIBILITY

Microsatellite genotypes: Zenodo https://doi.org/10.5281/zenodo.1042513

## AUTHOR CONTRIBUTION

HM, C.F. and P.G. designed research. H.M., C.F. and L.B. performed sampling. J.B. provided laboratory assistance. P.G. conducted molecular work and performed analyses. P.G., H.M. and C.F. wrote the manuscript.
