# Supplementary material for: From population connectivity to the art of striping Russian dolls: the lessons from Pocillopora corals
Source: Ecol Evol. 2017 Dec 27;8(2):1411–26. doi: 10.1002/ece3.3747 (PMC5773318; doi:10.1002/ece3.3747)
Supplement: Supplementary file 1 [file ECE3-8-1411-s001.pdf]

Appendix S1. Summary statistics of the 13 microsatellite loci used to genotype *Pocillopora* colonies assigned to PHS09 (*sensu* Gélin et al., 2017b). The number of alleles per locus (*Na*) and the number of private alleles per locus (*Np*) for the three SSHs (SSH09a, SSH09b and SSH09c) are given. The total number of alleles (Total) and the mean number of alleles per locus and standard error (Mean ± se) are also indicated for each SSH.

**Locus**	***Na* (SSH09a)**	***Np* (SSH09a)**	***Na* (SSH09b)**	***Np* (SSH09b)**	***Na* (SSH09c)**	***Np* (SSH09c)**	**Source**
**Pd2-001**	12	1	9	0	13	3	Starger *et al.* (2007)
**Pd3-004**	12	1	14	1	17	3	Starger *et al.* (2007)
**Pd3-005**	19	0	18	4	20	2	Starger *et al.* (2007)
**Pd2-006**	13	0	16	5	11	1	Starger *et al.* (2007)
**Pd3-008**	9	1	8	2	6	0	Starger *et al.* (2007)
**Pd3-009**	12	2	12	2	10	0	Starger *et al.* (2007)
**Poc40**	8	0	13	3	10	1	Pinzón & LaJeunesse (2011)
**PV2**	13	4	8	2	11	2	Magalon *et al.* (2004)
**PV7**	13	1	14	1	15	2	Magalon *et al.* (2004)
**Pd4**	21	4	12	1	13	2	Torda *et al.* (2013)
**Pd11**	19	3	14	1	16	0	Torda *et al.* (2013)
**Pd13**	22	4	15	2	21	3	Torda *et al.* (2013)
**Pd3-EF65**	15	1	16	0	16	0	Gorospe & Karl (2013)
**Total**	**189**	**22**	**169**	**24**	**179**	**19**	
**Mean ± se**	**14.54 ± 1.27**	**1.69 ± 0.43**	**13.00 ± 0.87**	**1.85 ± 0.41**	**13.77 ± 1.18**	**1.46 ± 0.33**	
