# Supplementary material for: From population connectivity to the art of striping Russian dolls: the lessons from Pocillopora corals
Source: Ecol Evol. 2017 Dec 27;8(2):1411–26. doi: 10.1002/ece3.3747 (PMC5773318; doi:10.1002/ece3.3747)

Appendix S2. Distribution of the allelic frequencies for PSH09. (a) Allelic frequencies for each locus for the three identified SSHs (SSH09a, SSH09b, SSH09c) and (b) Weir and Cockerham (1984) F_{ST} estimated per locus over all PSH09 colonies.

(a)

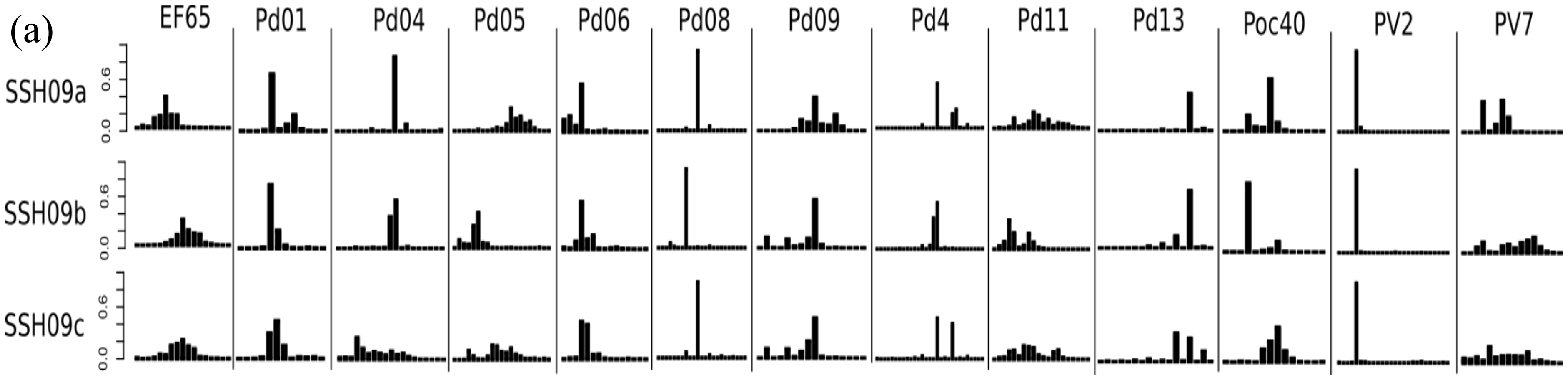

(b)

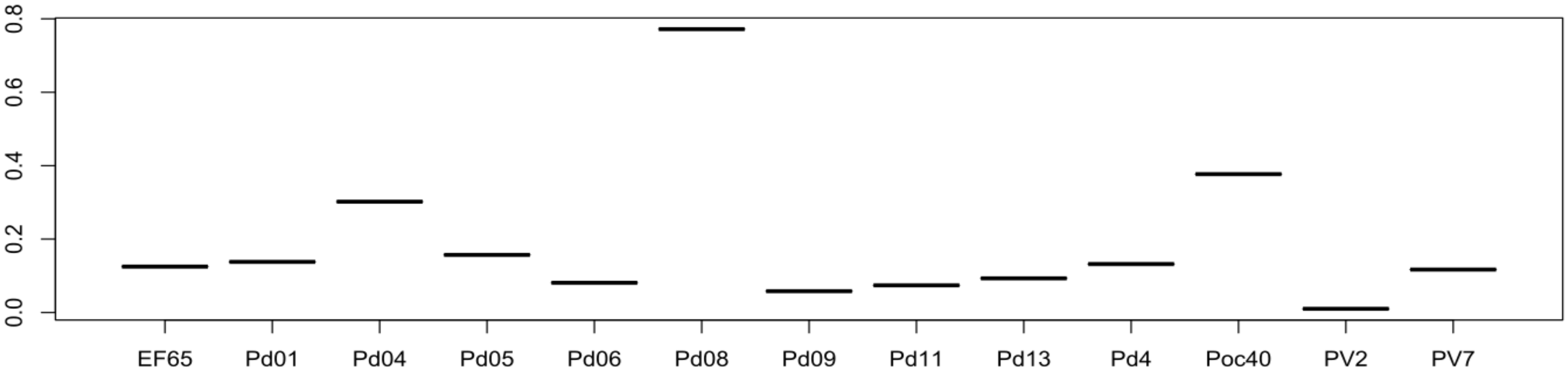

Supplement: Supplementary file 2 [file ECE3-8-1411-s002.pdf]
