# Supplementary material for: From population connectivity to the art of striping Russian dolls: the lessons from Pocillopora corals
Source: Ecol Evol. 2017 Dec 27;8(2):1411–26. doi: 10.1002/ece3.3747 (PMC5773318; doi:10.1002/ece3.3747)

Appendix S5. Distribution of the allelic frequencies for SSH09c. (a) Allelic frequencies for each locus for the three identified clusters (SSH09c-1, SSH09c-2, SSH09c-3) and (b) Weir and Cockerham (1984) F_{ST} estimated per locus over all SSH09c colonies.

(a)

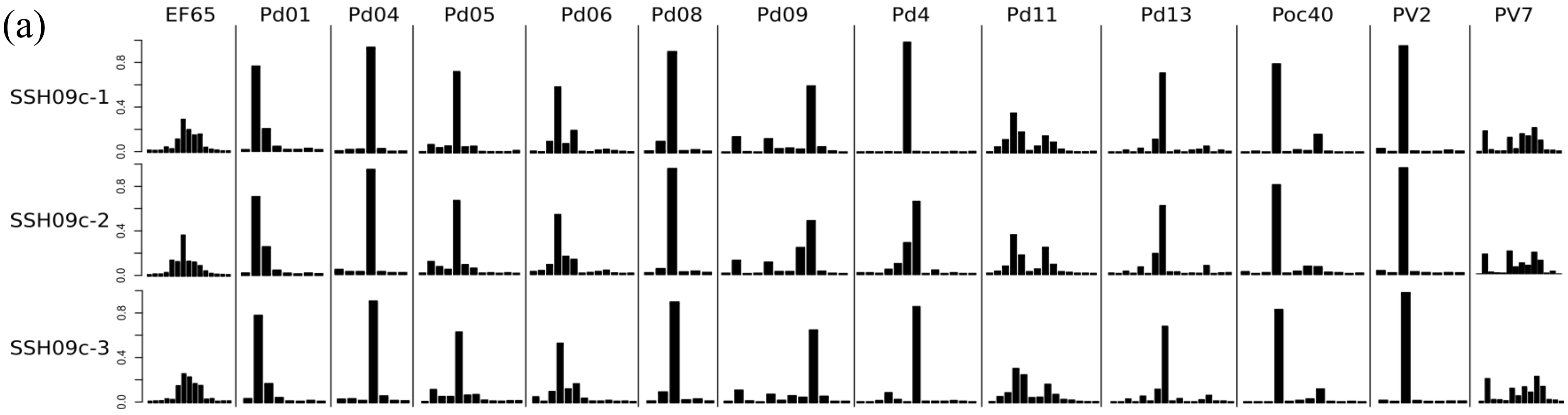

(b)

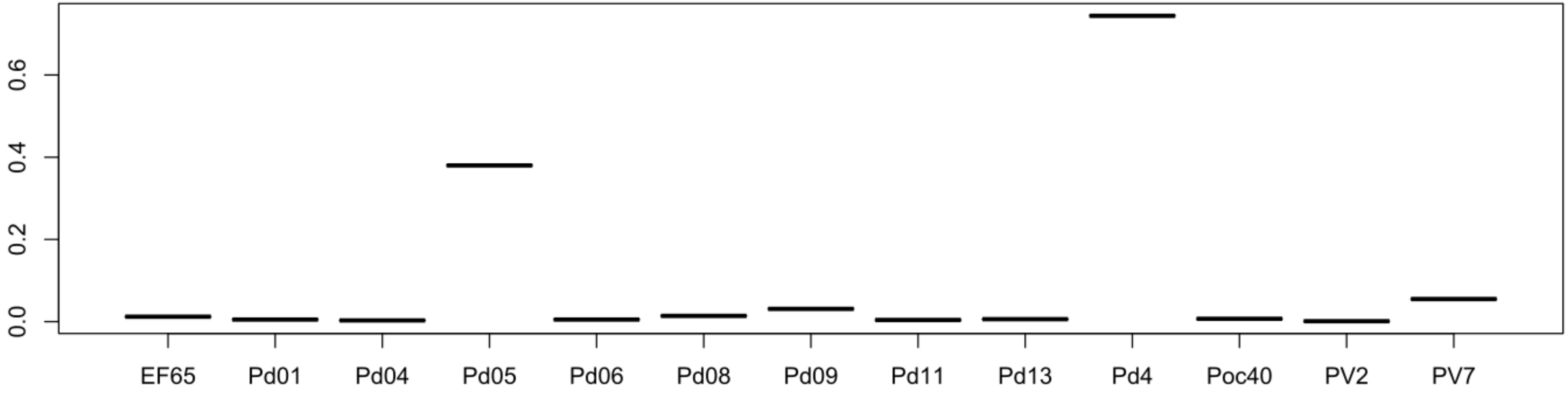

Supplement: Supplementary file 5 [file ECE3-8-1411-s005.pdf]
