# Supplementary material for: From population connectivity to the art of striping Russian dolls: the lessons from Pocillopora corals
Source: Ecol Evol. 2017 Dec 27;8(2):1411–26. doi: 10.1002/ece3.3747 (PMC5773318; doi:10.1002/ece3.3747)
Supplement: Supplementary file 6 [file ECE3-8-1411-s006.pdf]

Appendix S6. Analyses of molecular variance (AMOVA) of PSH09 colonies grouped according to the three SSHs identified and the corresponding clusters for each SSH. d.f.: degrees of freedom

Source of variation	d.f.	% of variation	*P*
**Among SSHs**	2	14.94	< 0.0001
**Among clusters within SSHs**	5	12.16	< 0.0001
**Among clusters**	3789	72.91	< 0.0001
